# Establishment of a rabbit liver metastasis model by percutaneous puncture of the spleen and implantation of the VX2 tumor strain under CT guidance

**DOI:** 10.1038/s41598-022-26706-w

**Published:** 2023-02-16

**Authors:** Bing Li, Guiling Feng, Lin Feng, Xu Feng, Qing Zhang, Chuan Zhang, Hanfeng Yang, Yong Du

**Affiliations:** 1 Department of Radiology, Affiliated Hospital of North Sichuan Medical College, 63 Wenhua Road, Nanchong, 637000 Sichuan People’s Republic of China; 2 Department of Radiology, The Second People’s Hospital of YiBin, 96 North Street, Yibin, 644000 Sichuan People’s Republic of China; 3 Department of Ultrasound, Affiliated Hospital of North Sichuan Medical College, 63 Wenhua Road, Nanchong, 637000 Sichuan People’s Republic of China; 4 Department of Pain, Affiliated Hospital of North Sichuan Medical College, Nanchong, 637000 1 South Maoyuan Road, People’s Republic of China

**Keywords:** Zoology, Medical research, Cancer imaging, Cancer models

## Abstract

This study aimed to compare the feasibility, success rate, and safety of establishing a rabbit VX2 liver metastasis model by percutaneous splenic implantation under CT guidance and open splenic implantation of the VX2 tumor strain. Fifty-two New Zealand white rabbits were randomly divided into group A (the percutaneous puncture group) (n = 26) and group B (the laparotomy group) (n = 26). In group A, 26 New Zealand white rabbits were implanted with tumor strains by percutaneous splenic puncture under CT guidance. In group B, 26 New Zealand white rabbits were implanted with tumor strains in the spleen by laparotomy. After 2–3 weeks of implantation, both group A and group B underwent MRI to confirm tumor growth in the spleen and metastasis to the liver. Two experimental rabbits randomly selected from groups A and B were killed for pathological examination. The success rate, complication rate, and operation time in groups A and B were compared and analyzed. A total of 23 rabbits in group A were successfully induced, and the success rate was 88.5% (23/26). The average time of operation was 14.42 ± 3.26 min. A total of 22 rabbits in group B were successfully induced, and the success rate was 84.6% (22/26). The average time of operation in group B was 23.69 ± 5.27 min. There was no significant difference in the success rate of induction between the two groups (*P* > 0.05). The MRI manifestations of liver metastases were multiple nodular and punctate abnormal signal shadows in the liver. Hematoxylin–eosin (HE) staining showed a large number of tumor cells in the tumor area. CT-guided percutaneous splenic implantation of the VX2 tumor strain to establish a rabbit liver metastasis model is a minimally invasive and feasible inducing method. The success rate of this technique is not lower than that of open splenic implantation, with low incidence of complications, and short operation time.

## Introduction

The liver is one of the organs most prone to malignant tumor metastasis^1^. At the same time, for patients with advanced multiple liver metastases, there are some shortcomings in targeted therapy, immunotherapy or hepatic artery infusion chemotherapy (HAIC), and a large number of related studies are needed^2,3^. To better understand the mechanism and to improve the treatment effect of liver metastases, many studies have been performed using experimental animal models^4,5^. At present, there are two methods to establish a rabbit liver metastasis model using the VX2 tumor strain^6,6^: (1) implanting the VX2 tumor strain directly in the rabbit liver; (2) implanting the tumor strain in the spleen to establish subsequent liver metastasis^7,7^. Research shows that, compared with the method that implants the VX2 tumor strain directly in the rabbit liver, the biological characteristics of the rabbit liver metastasis model formed by splenic implantation are closer to the biological characteristics of human liver metastasis^9^, metastases from spleen to liver are more consistent with clinical model of gastrointestinal tumor metastasis to liver via portal vein approach; the aforementioned model can better mimic the whole process of tumor growth and metastasis; thus, it is a more ideal animal model^9^. However, to date, as reported in studies, almost all the inducing methods of splenic implantation have used a laparotomy^9^. This method has disadvantages, such as large trauma and higher mortality. The method of implanting tumor strain into target organ by an image-guided percutaneous puncture to establish the tumor model has been reported. This method is associated with less injury and a low complication rate^10^. However, as the rabbit spleen is relatively small and difficult to puncture, there is no report on percutaneous splenic implantation under CT guidance.

This study intends to explore the feasibility of CT-guided percutaneous splenic implantation of the VX2 tumor strain to establish a rabbit liver metastasis model, and to compare its feasibility, success rate, and safety with open splenic implantation of the VX2 tumor strain.

## Materials and methods

### Animals

This research was approved by the animal ethics committee of Affiliated Hospital of North Sichuan Medical College. This study is in accordance with ARRIVE guidelines. Sample size was calculated using free sample size calculating software G.Power (Franz, Universitat Kiel, Germany). A pilot study was conducted in 12 rabbits, 6 rabbits received percutaneous puncture and the other 6 received laparotomy. The calculated effect size was 0.5. With α = 0.05, and a power of 0.95. Total sample size was calculated to be 52. A total of 52 healthy New Zealand white rabbits, aged from four to six months and weighing from 2.5 to 3.5 kg, were included in this study, and they were provided by the Experimental Animal Center, North Sichuan Medical College. And housed in animal center under controlled temperature (22 ± 2 °C) and humidity (40–60%), and they were provided with food and water ad libitum. To adapt to the environment, we kept healthy New Zealand white rabbits in our lab for at least one week, and they are more suitable for our following experiment. The rabbits were randomly divided into group A (the percutaneous puncture group) (n = 26) and group B (the laparotomy group) (n = 26).

### Preparation of VX2 tumor suspension

**Figure 1:**
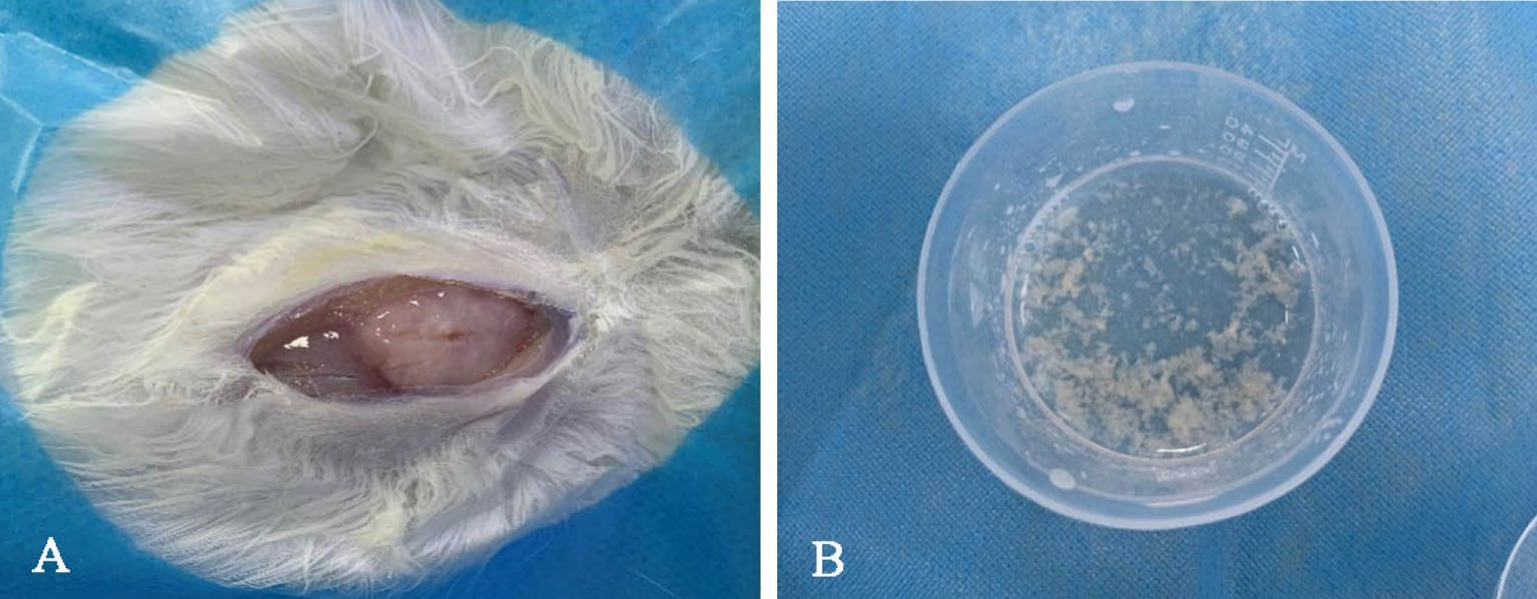
Preparation of VX2 tumor suspension. The tumor was removed from hind limb muscle of the rabbit (**A**); the VX2 tumor suspension was made (**B**).

A total of 30 mg/Kg of 3% sodium pentobarbital was injected into a VX2 tumor-bearing rabbit (purchased from Hangzhou Huashu Biotechnology Co., LTD) through the auricular vein. VX2 tumor-bearing rabbit was 14-week-old, and the VX2 tumor has been implanted into right hind limb muscle of the rabbit at 12 weeks of age. As previously described by Kageyama^4^ and Tabuchi et al.^9^ with slight modifications. The tumor was removed in a sterile environment; the necrotic tissue, fascia, blood vessels, and other tissues were also removed; only the white and transparent fish like tumor tissue was selected; the tissue has been crushed into small particles which diameter was not greater than 0.3 mm, and placed in normal saline to make a VX2 tumor suspension, the ratio of tumor tissue to normal saline was 1:3 (Fig. 1).

### CT guided percutaneous splenic implantation

**Figure 2:**
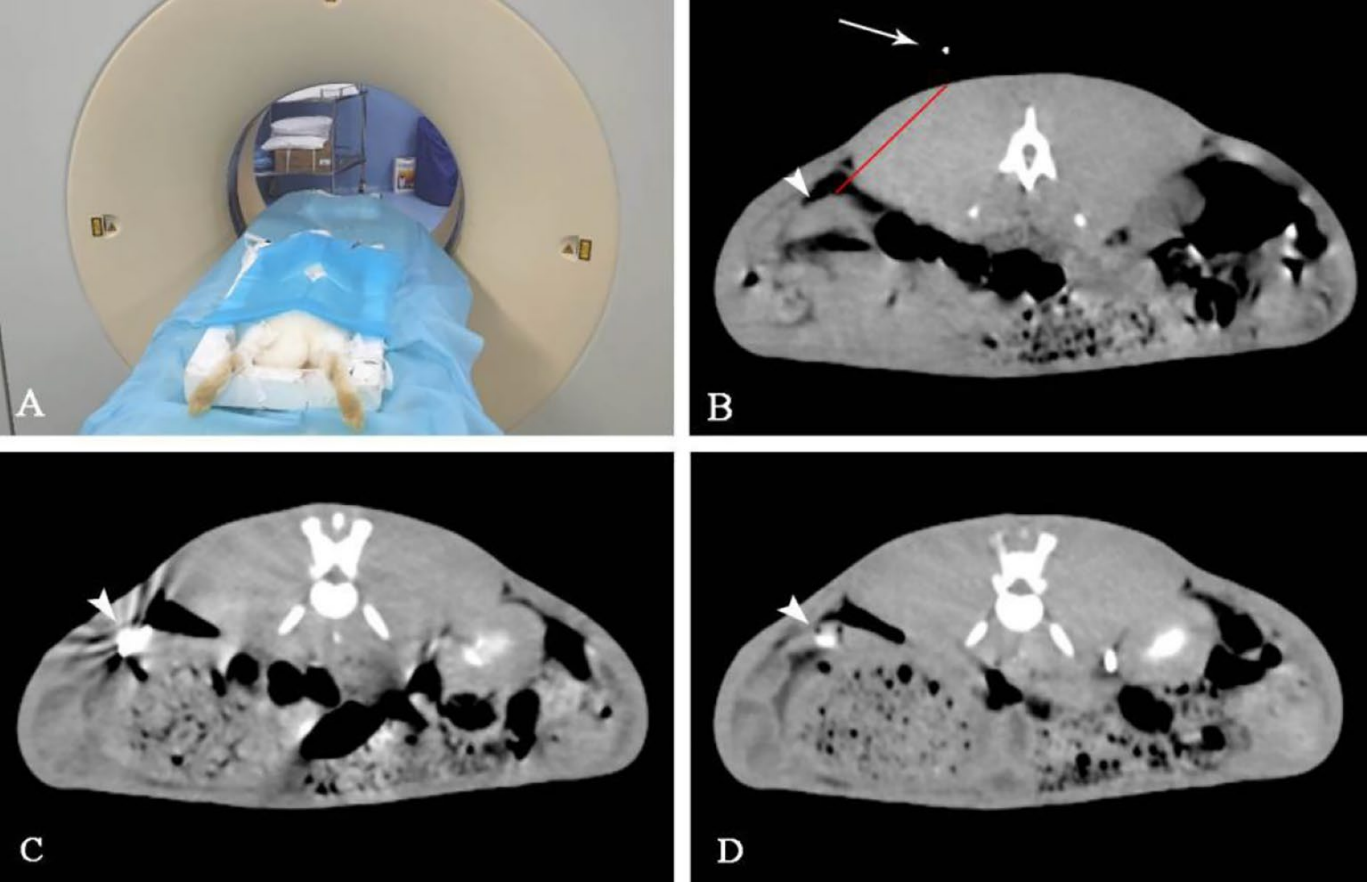
CT-guided percutaneous splenic implantation. The rabbits were fixed on the CT scanning table in a prone position (**A**); A self-made metal positioner (arrow) was used for positioning, the puncture path was determined (red line) (**B**), spleen (arrowhead); a 22G coaxial puncture needle (arrowhead) was used to puncture the rabbit spleen (**C**); The needle path was blocked with a gelatin sponge (arrowhead) (**D**).

After general anesthesia, the rabbits were fixed on the CT scanning table in a prone position. All operations are performed by the same experienced interventional physician (Bing Li, with 10 years experience). CT scanning was performed firstly to find the spleen, and the self-made metal positioner was placed on the left side of the spinous process at the spleen level. Then, CT scanning was performed again to determine the puncture path. According to the location of positioner and spleen, the puncture path, direction, and depth were determined. An oblique puncture angle from the inside to the outside via the paraspinal musculature should be formulated. Under sterile conditions, a 22G coaxial puncture needle (Hakko Medical, Japan) was used to puncture the rabbit spleen step by step under CT guidance. The needle was inserted into the dorsal muscles of the rabbit firstly, and CT scanning was given to confirm the position of the needle, and then the needle was punctured into the inside of the spleen. After confirmation of the position of the needle tip by CT scanning, the VX2 tumor suspension (about 0.1–0.2 ml) was injected into the spleen. After dropping the suspension, the needle path was blocked with a gelatin sponge with contrast. The needle was pulled out and the puncture point was pressed for 1–2 min. CT scanning was performed to determine whether there were complications such as bleeding, when perisplenic or peritoneal effusion appears on CT scanning after operation, considered that there was bleeding (Fig. 2). Postoperatively, rabbits were kept warm with a heater and returned to their cages when they became fully awake. All animals received penicillin (0.4 MU, q.d, IM) and Meloxicam (0.2 mg/KG q.d SC) for 3 consecutive days after the operation.. And the adverse events such as pain and decreased appetite are also recorded. The pain assessment of rabbits referred to the method of composite pain scale for rabbit (CANCRS)^11^.

### Open splenic implantation

After general anesthesia, the rabbits were fixed on the experimental table in a supine position and they were depilated through the lower edge of the left costal arch. The depilated area was then disinfected locally, and a 3-cm incision was made with a scalpel to expose the spleen. The VX2 tumor suspension was then injected into the exposed spleen with a 22G needle, then pressed the puncture point. The abdominal wall was closed after no fluid and blood outflow from the spleen was confirmed. CT scanning was performed to determine whether there were complications such as bleeding. The abdominal incision was closed with suture manual technique. Postoperatively, rabbits were kept warm with a heater and returned to their cages when they became fully awake. All animals received penicillin (0.4 MU q.d IM) and Meloxicam (0.2 mg/KG q.d SC) for 3 consecutive days after the operation.. The pain and decreased appetite of the rabbits are also recorded.

### Magnetic resonance scanning and sequence parameters

According to the pre-experimental results and the experience of other relevant literatures, liver metastatic tumors will grow to 1.5–2 cm in 2–3 weeks^7,7^. Therefore, we performed Magnetic Resonance Imaging (MRI) scanning at 2–3 weeks after implantation. Both groups A and B underwent MRI scanning (SIEMENS AG, Aero) to confirm tumor growth in the spleen and metastasis to the liver. A 15-channel knee phase-controlled front coil was used. After general anesthesia, rabbit liver metastasis models were placed in the channel coil for a conventional MRI scan, and the scan sequence included 3D-Vibe-T1WI, FS-TSE-T2WI, diffusion-weighted imaging (DWI), and 3D-Vibe-T1WI gadodiamide-enhanced MRI scan (Table 1). If no lesion was found on MRI scanning, the last scanning was performed at the 4th week after operation. MRI findings were interpreted by a senior radiologist (Yong Du, with 36 years experience).

**Table 1:** MR scanning and sequence parameters.

MR sequence	Patient position	TE(ms)	TR(ms)	Slice thickness (mm)	Matrix	FA	FoV(mm)
fs-tse-T2WI	axial	110.0	3000.0	3.0	256 × 256	140.0	180 × 180
3D-Vibe-T1WI	axial	5.0	10.0	1.2	320 × 195	10.0	260 × 211
DWI	axial	77.0	6000.0	3.0	112 × 112	90.0	230 × 230
3D-Vibe-T1WI + C	axial	5.0	10.0	1.2	320 × 195	10.0	260 × 211

*TE* echo time; *TR* repetition time; *FA* flip angle; *FoV* field of view.

### Histological observation

Two experimental rabbits were randomly selected from groups A and B, and they were killed by injecting 100 mg/Kg of 3% sodium pentobarbital along the ear vein. The spleen and liver were removed under sterile conditions. The spleen tumor and liver metastasis were identified, and the location, shape, size, color, and boundary of the tumor were observed. The tumor was peeled out, cut into small pieces, and soaked in 10% neutral formaldehyde fixation solution; then the fixed tumor was dehydrated and sliced, and the pathological changes were observed. The histopathological observation was performed by one experienced pathologist (with 24 years experience). Remaining rabbits have been used for subsequent experiments, so the histopathology was not performed in these animals.

### Statistical analysis

Statistical Package for Social Sciences software (version 23.0, IBM) was used for data statistical analysis. The mean ± standard deviation ($\overline{x}$  ± s) was used for all measurement data, and a chi-square test (χ2 test) was used for the count data. The success (defined as presence of intrahepatic lesions without needle track and peritoneal metastasis) rate of inducing, operation time, complication rate, and adverse events in the two groups were compared and analyzed. *P* < 0.05 was considered statistically significant.

### Statement

This study was approved by the Animal Ethics Committee of North Sichuan Medical College [Approval No. : NSMC Ethical Animal Review (2021)51], and a total of 50 New Zealand white rabbits were included in the experimental study. New Zealand white rabbits were provided by Experimental Animal Center of North Sichuan Medical College [License Number: SYXK (Sichuan) 2018–076], with body weight of 2.5 ~ 3.5 kg, age of 4 ~ 6 months, male only, with health quarantine certificate. All experiments were performed in accordance with relevant guidelines and regulations.

## Results

### Inducing of rabbit liver metastasis

A total of 23 rabbits in group A were successfully induced, and the success rate was 88.5% (23/26). One of the rabbits had no tumor growth, one had both needle track and peritoneal metastasis, and one had only peritoneal metastasis. The average time of tumor operation (from the time of the first CT imaging to the time of the post-procedure confirmatory CT imaging) was 11.19 ± 3.11 min. A total of 22 rabbits in group B were successfully induced, and the success rate was 84.6% (22/26). Two of the rabbits died within 3 weeks. After death, one rabbit was found to have multiple peritoneal metastases and a large amount of peritoneal effusion, but there was no obvious metastasis in the liver. The cause of death of another rabbit was not known, and the other two rabbits had peritoneal metastasis. The average time of tumor operation (from incision of skin to suture completion) in group B was 23.69 ± 5.27 min. No obvious complications (such as bleeding, infection, gastrointestinal tract injury, ascites) occurred in group A. There were 4 cases of skin wound infection in group B, no other complications. There were significant differences in the operation time and complication rate between the two groups (*P* < 0.05). But there was no significant difference in the success rate of inducing and dissemination rate between the two groups (*P* > 0.05) (Table 2). And the average number of punctures of group A were 2.81 ± 0.85, the average number of CT scanning were 4.88 ± 1.03.

**Table 2:** Comparison of rabbit VX2 liver metastasis inducing in groups A and B.

Parameter	Group		*P* value
	Percutaneous puncture (A)	Laparotomy (B)	
Rabbit (No.)	26	26	
Success rate (%)	88.5	84.6	0.692
Operation time (min)	14.42 ± 3.26	23.69 ± 5.27	< 0.001
Complication rate (%)	0	15.38	0.043
Dissemination rate (%)	7.69	11.54	0.646
Number of deaths	0	2	0.161
**Adverse events**			
*Pain*			
*No pain*	24	18	0.036
*Discomfort*	1	6	0.045
*Moderate Pain*	1	2	0.561
*Severe Pain*	0	0	/
*Decreased appetite*	6	8	0.541
Maximum diameters (mm)	15.34 ± 4.52	14.92 ± 4.20	0.728
Number of punctures	2.81 ± 0.85	/	/
Number of CT scanning	4.88 ± 1.03	/	/

Success was defined as presence of intrahepatic lesions without needle track and peritoneal metastasis; Dissemination: including needle track and peritoneal metastasis.

### MRI findings

**Figure 3:**
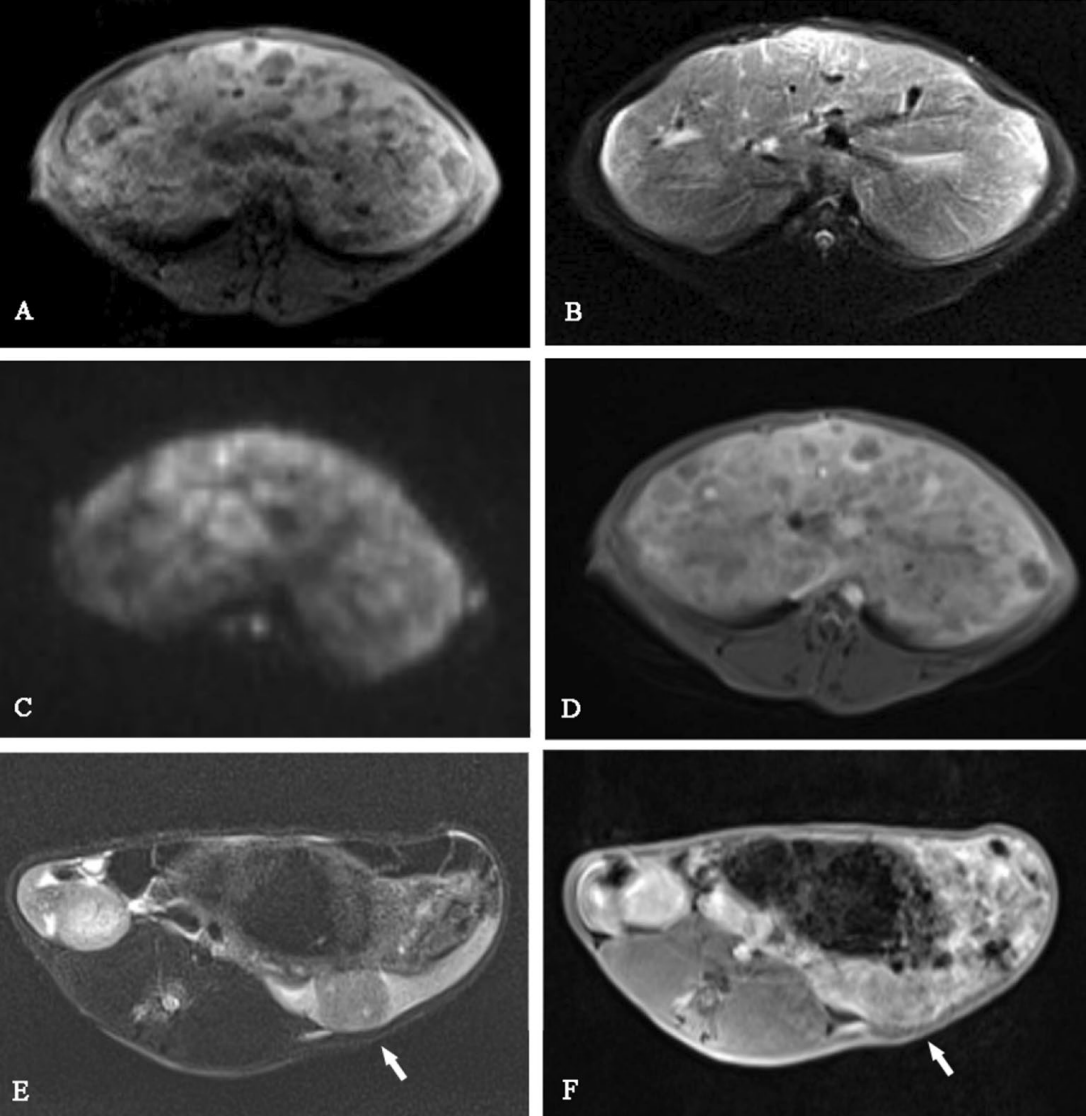
MRI manifestations of liver metastases were multiple nodular and punctate abnormal signal shadows in the liver, low signal on T1WI (**A**), slightly high or equal signal on T2WI (**B**), high signal on DWI (**C**), and circular enhancement on enhanced scanning (**D**); E, F: T2WI and T1WI sequences showed that the liver was enlarged and with a nodular shadow (white arrow).

MRI manifestations of liver metastases were multiple nodular and punctate abnormal signal shadows in the liver, low signal on T1WI, a slightly high or equal signal on T2WI, and a high signal on DWI. Circular enhancement was seen on enhanced scanning, and no enhancement area was seen in the center. The specimens showed that liver metastases were multiple round-like nodules, the edges were irregular, and the boundary with the normal liver tissue was still clear (Fig. 3). The maximum diameters of liver metastases of group A and group B were 15.34 ± 4.52 mm and 14.92 ± 4.20 mm respectively (Table 2). The MRI findings of the liver metastases of two groups are similar.

### Pathological findings

**Figure 4:**
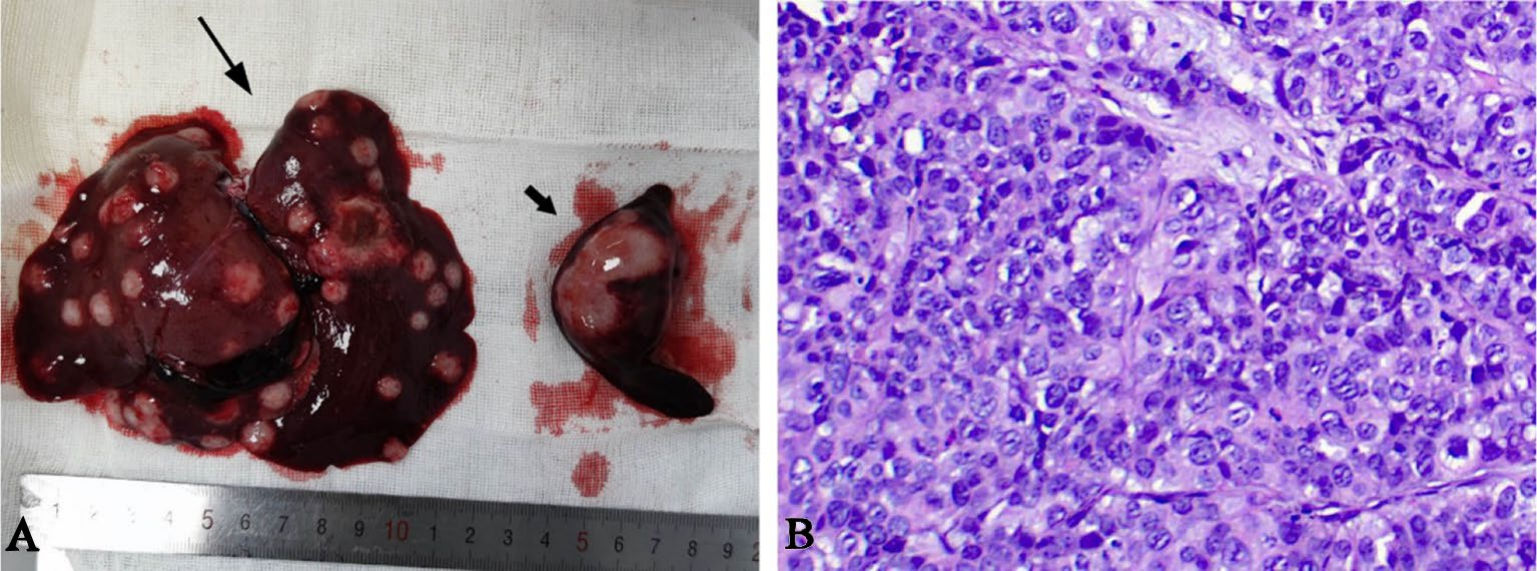
The specimens (**A**) showed multiple gray-white nodular shadows in the liver (long black arrow); The volume of the spleen (short black arrow) was increased, and a fish-like nodular tumor could be seen; (**B**) HE staining showed that the nucleus was large and deeply stained, and the morphology was irregular.

The tumor was gray, transparent, and fish-like, with dotted milky white necrotic areas in the middle. The spleen tumor was massive or nodular, grayish-white and transparent fish-like, and the volume of the spleen was increased. Hematoxylin–eosin (HE) staining showed a large number of tumor cells in the tumor area, the tumor cells had large, dark-stained nuclei and an irregular morphology, and a large number of necrotic cells were located in the center of the tumor (Fig. 4) . There was no difference in the pathology findings between two groups.

## Discussion

The establishment of an animal liver metastasis model has great significance for clinical and scientific research^8,12^. In this study, a rabbit liver metastasis model was established by percutaneous splenic implantation of the VX2 tumor strain under CT guidance, and it was compared with the establishment of open splenic implantation. The results showed that CT guided percutaneous splenic implantation of the VX2 tumor strain to establish a rabbit liver metastasis model is a minimally invasive and feasible method, the success rate of this method is not lower than the method using open splenic implantation, and this method is associated with low incidence of complications, and short operation time. At present, a similar method has not been reported.

The study showed that the biological characteristics of the rabbit liver metastasis model formed by splenic implantation are closer to the biological characteristics of human liver metastasis^13^. Shi Bo et al.^7^ reported that the model established by spleen inoculated with the VX2 tumor strain was found to be closer to human primary liver metastasis by using the CT scan and digital subtraction angiography (DSA). Our research has attained the similar results. But laparotomy can cause large trauma, and then can lead to higher mortality^7^. One study has also reported the use of an image-guided percutaneous puncture target organ to establish a tumor model^14^. But the rabbit spleen is relatively small, and it is difficult to operate under ultrasound guidance. Therefore, we established a liver metastasis model by puncturing the spleen under CT guidance.

The results of this study showed that CT-guided percutaneous splenic implantation to establish a rabbit liver metastasis model is a feasible inducing method, and its success rate is similar to that of open splenic implantation^7^. In group A, one rabbit had no tumor growth, one had both needle track and peritoneal metastasis, and one had only peritoneal metastasis. The reason for this occurrence may be that the needle tip did not enter the spleen during the drip of VX2 tumor suspension; thus, the VX2 tumor strain was implanted in the peritoneum and puncture path. There were 3 cases of peritoneal metastasis in group B, of which 2 rabbits died. The reason for this occurrence may be that the VX2 tumor suspension leaked along the puncture path after implanting into the spleen, resulting in extensive peritoneal implantation. Ozaki et al. cauterized the insertion site of the needle in the mice liver to prevent the leakage of cell suspension and bleeding, and achieved a good result, this method is also recommended when use open splenic implantation^15^. On the comparison of complications, four cases of wound infection occurred in group B, as the laparotomy is more traumatic. No obvious complications occurred in group A. The result indicates that the method of CT-guided percutaneous puncture is associated with less trauma and a low incidence of complications.

Attention should be paid to the following aspects in the operation of CT-guided percutaneous splenic implantation. (1) Localization scanning: because the rabbit spleen is small, thin-layer continuous scanning with a thickness of 1–2 mm is required, and the largest slice of the spleen should be selected for puncture. In addition, the spleen is crescent-shaped; thus, we should formulate an oblique puncture angle from the inside to the outside, increase the length of the needle path in the spleen, and then reduce the probability of penetrating the spleen to damage the stomach and other structures. (2) Needle selection: due to the small volume and large mobility of the rabbit spleen, a 22G coaxial puncture needle should be selected, as the needle tip is inclined and sharp, which can reduce the probability that the use of a small force cannot puncture the spleen and use of a large force completely penetrates the spleen, and it can reduce the risk of spleen rupture and bleeding. (3) Puncture technology: when the needle tip approaches the spleen, it is necessary to quickly puncture the spleen, which can effectively reduce the repeated puncture and save the puncture time. (4) After dropping the suspension, the needle path should be blocked with a gelatin sponge to prevent the leakage of tumor suspension, which can reduce the incidence of peritoneal and needle path metastases.

Meanwhile, the operation time of CT-guided percutaneous splenic implantation is significantly lower than that of open splenic implantation. After mastering the key points of CT-guided percutaneous splenic implantation, the operator can complete the operation in a short time. However, the steps of laparotomy are relatively complex and cumbersome. If the laparotomy incision is small, it is difficult to identify the spleen; and if the incision is large, the injury to the rabbit is obvious and it also increases the risk of infection^13^. A skin suture and other operations are also required; thus, a relatively long time is needed. At the same time, the number of dead rabbits in group B were also higher than that in group A, although the difference was not statistically significant.

MRI manifestations of the two groups of liver metastases were multiple nodular-like abnormal signal shadows in the liver, low signal on T1WI, a slightly high or equal signal on T2WI, high signal on DWI, and circular enhancement on enhanced scanning. They were typical imaging manifestations of liver metastasis, which were similar to those of human liver metastasis^16–18^. In addition, HE stained sections from groups A and B showed that there were vigorous tumor cells in liver metastases. There was no difference in the imaging and pathological findings between the two groups. This finding is consistent with the research by Shi Bo et al^7^.

There are also some limitations in this study. No centrifuge was used for the preparation of tumor suspension, and the cell concentration was not quantified, although the success rate of inducing is high. And the sample size of this study has just reached the statistical requirements, the large sample size study needs to be further carried out. Another limitation is that the complication is not confirmed by any means other than CT imaging.

## Conclusion

In this study, CT-guided percutaneous splenic implantation of the VX2 tumor strain was used to establish a rabbit liver metastasis model, which is a minimally invasive and feasible inducing method. The success rate of the use of the aforementioned model was similar to that of open splenic implantation, and is associated with low incidence of complications, and short operation time. Furthermore, the proposed method provides a new and minimally invasive modeling method for clinical use, which has important clinical and scientific research significance.

## Data Availability

Raw data obtained and analyzed from this study are available from the corresponding author upon reasonable request.

## Abbreviations

CT: Computer tomography
MRI: Magnatic Resonance Imaging
HE: Hematoxylin–eosin
